# Unenhanced Cardiac Magnetic Resonance may improve detection and prognostication of an occult heart involvement in asymptomatic patients with systemic sclerosis

**DOI:** 10.1038/s41598-022-09064-5

**Published:** 2022-03-24

**Authors:** Pierpaolo Palumbo, Piero Ruscitti, Ester Cannizzaro, Onorina Berardicurti, Alessandro Conforti, Annamaria Di Cesare, Ilenia Di Cola, Roberto Giacomelli, Alessandra Splendiani, Antonio Barile, Carlo Masciocchi, Paola Cipriani, Ernesto Di Cesare

**Affiliations:** 1 Department of Diagnostic Imaging, Area of Cardiovascular and Interventional Imaging, Abruzzo Health Unit 1, Via Saragat -località Campo di Pile, 67100 L’Aquila, Italy; 2 SIRM Foundation, Italian Society of Medical and Interventional Radiology (SIRM), 20122 Milan, Italy; 3 Department of Biotechnological and Applied Clinical Sciences, University of L’Aquila, Via Vetoio 1, 67100 L’Aquila, Italy; 4 Ospedale “Infermi” di Rimini, Viale Luigi Settembrini, 2, 47923 Rimini, Italy; 5 Rome Biomedical Campus University, via Álvaro del Portillo 200, 00128 Roma, Italy; 6 Department of Life, Health and Environmental Sciences, University of L’Aquila, Piazzale Salvatore Tommasi 1, 67100 L’Aquila, Italy

**Keywords:** Cardiology, Rheumatology, Rheumatic diseases, Diagnostic markers, Predictive markers

## Abstract

Systemic sclerosis (SSc) is an uncommon autoimmune disease. Aim of the study was to detect the occult cardiac involvement in asymptomatic SSc patients of recent onset (indicative of a more aggressive disease) with unenhanced Cardiac Magnetic Resonance (CMR). Our historical prospective study included naïve SSc patients of recent onset. Modified Rodnan Skin Score (mRSS) and Scleroderma Clinical Trial Consortium Damage Index (SCTC-DI) were calculated. Cardiac volumes and global myocardial strain were assessed and also compared with healthy group values. Pericardial involvement was further recorded. Thirty-one patients met inclusion criteria (54 ± 12 years; 1 M). Mean duration of disease was 6.8 years. All patients showed preserved systolic function. Higher incidence of pericardial involvement was founded in patients with disease accrual damage (OR: 9.6, *p*-value 0.01). Radial and longitudinal strain values resulted significantly different between healthy and SSc patients. GRS and GLS showed an independent predictive validity on damage accrual (HR: 1.22 and 1.47, respectively). Best C-index for disease progression was reached when strain values and pericardial evaluation were added to conventional risk factors (0.97, *p*-value: 0.0001). Strain analysis by CMR-TT may show a high capability both in identifying early cardiac involvement and stratifying its clinical aggressiveness, regardless of the standard damage indices and CMR contrast-dependent biomarker.

## Introduction

Systemic sclerosis (SSc) is an autoimmune disease characterised by a widespread microangiopathy, autoimmunity, and aberrant fibrosis of skin and internal organs^1^.

SSc has a high impact on daily activity and a poor prognosis^2^. Clinically, SSc is a very heterogeneous disease and may generally be classified into two different forms based on the extent of skin involvement, either diffuse cutaneous sclerosis (dcSSc) or limited cutaneous sclerosis (lcSSc)^4^. These different SSc subtypes differentiate in pattern and organ pathology, disease progression and outcomes.

However some patients do not fit neatly into these subclasses^3^ and a Very Early Diagnosis Of Systemic Sclerosis or VEDOSS subset has been proposed, remaining of difficult diagnosis due to low sensitivity of current criteria^4,5^. Moreover, there is still no agreement on the predictors that may allow to identify patients who will progress form VEDOSS to a definite disease^6^. These difficulties often lead to delay in diagnosis and proper treatment, often carry out when internal organ involvement has already irreversible.

Detection of valid predictors of disease damage since the earlier stages remains therefore mandatory^1^. An early predictor of disease evolution could offer the opportunity for a timely and effective treatment of SSc organ damage^7,8^.

In this context, it has been shown that SSc heart disease (HD), affecting the endocardium, myocardium, pericardium and cardiac conduction system, separately or concomitantly, is associated with a poor prognosis and increased mortality when clinically manifest^9,10^. Thus, a growing body of evidence is focused on unmasking an occult SSc-HD in order to provide a proper management of those patients^11^.

In the last years, more and more accurate techniques have been proposed to be more sensitive methods than conventional echocardiography to allow a very early diagnosis and a possible timely treatment^12,13^. On these bases, main aims of our study were to detect an occult cardiac involvement in asymptomatic SSc patients of recent onset with unenhanced Cardiac Magnetic Resonance (CMR), assessing the evolution of disease in light of a very early myocardial involvement.

## Material and methods

### Patients and setting

In this study, we performed a retrospective analysis of prospectively followed-up patients with SSc attending our Rheumatologic Clinic, between January 2010 and December 2020. Our patient population included naïve SSc patients with recent onset of disease (fulfilling the ACR/EULAR 2013 classification criteria), in less than 1 year from the onset of Raynaud’s phenomenon, submitted to a resting CMR and deemed able to complete a long-term follow-up or VEDOSS patients^7,14^. All patients with SSc were properly classified as lcSSc or, dcSSc, according to previous criteria^15^. A healthy control group was also recruited (23 participants; 12 males, 44 ± 9 years), based on the absence of structural heart diseases (regular ventricular volumes and geometry, preserved EF and absence of valvular disease) or positive late gadolinium enhancement (LGE) findings and evidence of regular volumes and function. The healthy controls were recruited among those people referred to our center for echocardiographic suspicion of cardiomyopathy but not confirmed with CMR, in absence of a clinical history of myocardial injury and/or systemic disease.

The local ethics committee approved the study protocol, which has been performed according to the Good Clinical Practice guidelines and the Declaration of Helsinki.

### Exam protocol

CMR exams were performed with a 1.5-T (GE Signa Horizon HD).

For the assessment of LV volumes, function, and myocardial mass, steady-state free precession cine images (echo time/reception time 1.5/3.0 ms, flip angle 60°) were acquired on short-axis (slice thickness 8 mm, spacing 0 mm) and radial long-axis views (i.e., ten slices covering the entire circumference of the ventricle, planned on short-axis pilots at 18° angles to each other to visualize all 17 segments, according to the American Heart Association recommendations).

LGE was excluded from the current analysis (please, refer to limit section).

### Post-processing analysis

Post-processing analysis was performed with a dedicated software (Circle, cvi^42^, Calgary, Canada; version 5.11.4). Endocardial contours were manually drawn during both diastolic and systolic phases to evaluate LV volumes and systolic function. Epicardial contours were defined in the diastolic phase to evaluate myocardial mass^16^. Volumes were collected as absolute and indexed values.

Tissue Tracking analysis also was performed. In the TT analysis, the diastolic phase was contoured and corrected after registration for any potential bias. Left ventricular outflow tract and mitral valve planes were excluded from the analysis. 2D values were preferred to 3D values, for potential mismatch between short and long-axis.

Global 2d longitudinal (GLS), circumferential (GCS), and radial (GRS) strain values were recorded.

Evidence of significant pericardial effusion (> 5 mm) and/or thickness (≥ 2 mm) suggestive for pericardial involvement were collected^17^.

All analysis were performed by a single reader with high expertise in CMR field.

### Clinical follow-up study

Systemic involvement was evaluated through both 23-items Scleroderma Clinical Trial Consortium Damage Index (SCTC-DI) and modified mRSS^18,19^. All patients were submitted to a clinical follow-up and both SCTC-DI and mRSS were calculated at baseline (time of recruitment) and during follow-up. Patients were categorized according SCTC-DI in low (< 5), moderate (6–12) and high damage (≥ 13). Clinically relevant endpoint considered the progression of disease defined as an increase of accrual of damage. In absence of a known cut-off, accrual of damage was considered if last assessed score progressed to a highest damage profile, meaning the change from a lower accrual damage category to a higher one. Presence of fibrotic manifestations, Pulmonary Artery Systolic Pressure (PASP), and presence of cardiovascular risk factor were also collected. Duration of disease was considered the time ranging from the first diagnosis to control.

### Statistical analysis

Descriptive variables are presented as mean and correspondent standard deviation or confidential intervals. When required by the analysis, some variables were categorized as dichotomous variables. The Shapiro–Wilk (SW) test was used to evaluate data distribution. Despite the small sample size, the distributional assumption for parametric analysis is fulfilled according to the SW test. T-test were used to evaluate the distribution of data among different subsets. A chi-square test was used for dichotomous variables.

Strain data were compared with healthy values, both in a global and per-subset analysis using a Kruskal–Wallis test for multiple comparison with a Bonferroni adjustment. Pearson product-moment r correlation was performed to evaluate potential correlation between strain and clinical variables. Regression analysis was performed for an interpretative/predictive evaluation of founded correlations.

Association and predictor validity of all variables was tested by univariate and multivariate Cox regression analysis. C-index for different predictive models estimated via Cox regression was performed. An alpha error of 5% was used as a threshold of significance. All statistical analyses were performed with SPSS (IBM Corp. Released 2016. IBM SPSS Statistics for Mac, Version 26.0. Armonk, NY: IBM Corp).

Graphs were obtained via GraphPad Prism (version 8.0.0, GraphPad Software, San Diego, California USA).

## Results

### Patient population

31 patients met inclusion criteria with a mean age of 54 ± 12 years. Only 1 patient was male. Cardiovascular risk factor were: (i.) Type-2 diabetes mellitus^1^; (ii.) hypertension^2^; (iii.) dyslipidemia^6^; (iv.) familiarity^4^; (v.) history of smoking habit^16^.

The patients were homogeneously distributed in the three subsets. Baseline characteristics of patient population are listed in Table 1. Notably, seven patients were categorized as moderate damage according to the baseline SCTC-DI score, while the remaining 25 patients were categorized as low damage. Different subset showed significant difference in mRSS, with high score in dcSSc subset.

**Table 1:** Patients characteristics.

	All (n. 31)	VEDOSS (n. 11)	LcSSc (n. 10)	DcSSc (n. 10)	*p*-value	*p*-value
**Baseline characteristics**						
Age (years)	54 ± 12	56 ± 12	49 ± 11	58 ± 11	0.169	
BMI (kg/m^2^)	23.5 ± 4	22.7 ± 3	24.8 ± 4	23.5 ± 3	0.67	
PASP (mmHg)	27.4 ± 10	23.5 ± 7	25.7 ± 7	32.9 ± 15	0.138	
SCTC-DI (baseline)	*3* ± *2*	*3* ± *2*	*4* ± *2*	*3* ± *3*	*0.796*	0.0001**
SCTC-DI (last)	*8* ± *4*	*6* ± *4*	*6* ± *3*	*9* ± *4*	*0.131*	
Damage Accrual	11	2	5	4	0.454	
mRSS (baseline)	*7* ± *7*	*3* ± *3*	*5* ± *4*	*12* ± *7*	0.004**	*0.724*
mRSS (last)	*7* ± *6*	*3* ± *4*	*5* ± *4*	*12* ± *7*	0.003**	
**CMR**						
LVEDV/BSA (ml/m^2^)	69 ± 13	72 ± 11	67 ± 18	68 ± 10	0.405	
LVSV/BSA (ml/m^2^)	41 ± 8	43 ± 7	39 ± 9	41 ± 9	0.707	
LVEF (%)	60 ± 7	60 ± 7	60 ± 8	61 ± 7	0.876	
MyoMass/BSA (g/m^2^)	55 ± 13	55 ± 14	57 ± 17	53 ± 10	0.998	
RVEDV/BSA (ml/m^2^)	70 ± 16	73 ± 14	68 ± 19	69 ± 16	0.716	
RVEF (%)	57 ± 7	56 ± 8	58 ± 7	58 ± 5	0.78	
Pericardial Involvement n.(%)	9 (29)	2 (6)	4 (13)	3 (10)	0.55	

VEDOSS: very early diagnosis of systemic sclerosis; LsSSc: Limited cutaneous Systemic Sclerosis; DcSSc: Diffuse cutaneous Systemic Sclerosis; BMI: body mass index; PASP: pulmonary artery systolic pressure; SCTC-DI: Scleroderma Clinical Trial Consortium Damage Index; mRSS: modified Rodnan Skin Score; CMR: cardiac magnetic resonance; LVEDV: left ventricular end-diastolic volume; BSA: body surface area; LVSV: left ventricular systolic volume; LVEF: left ventricular ejection fraction; MyoMass: myocardial mass; RVEDV: right ventricular end-diastolic volume; RVEF: right ventricular ejection fraction; SSc: systemic sclerosis.

**Significant test; *p*-value less than 0.01. In italic, SCTC-DI and mRSS were respectively paired for baseline and at last follow-up values. First p-value refers to the per subset analysis. In the last column, comparison between paired overall values.

Mean follow-up was 6.8 years (interquartile range: from 2 to 9). An increase of SCTC-DI score was observed in our cohort at the end of observation period, mostly in the patients with dcSSc. Accrual damage was evidenced in 11 patients, without significant difference between the three subsets.

All patients showed regular volumes and global function as in accordance with reference values available in literature (Table 1)^20^.

CMR-TT was performed in all patients, showing no significant difference between strain values categorized according to the three subsets.

### Strain analysis and comparison with the healthy group

**Figure 1:**
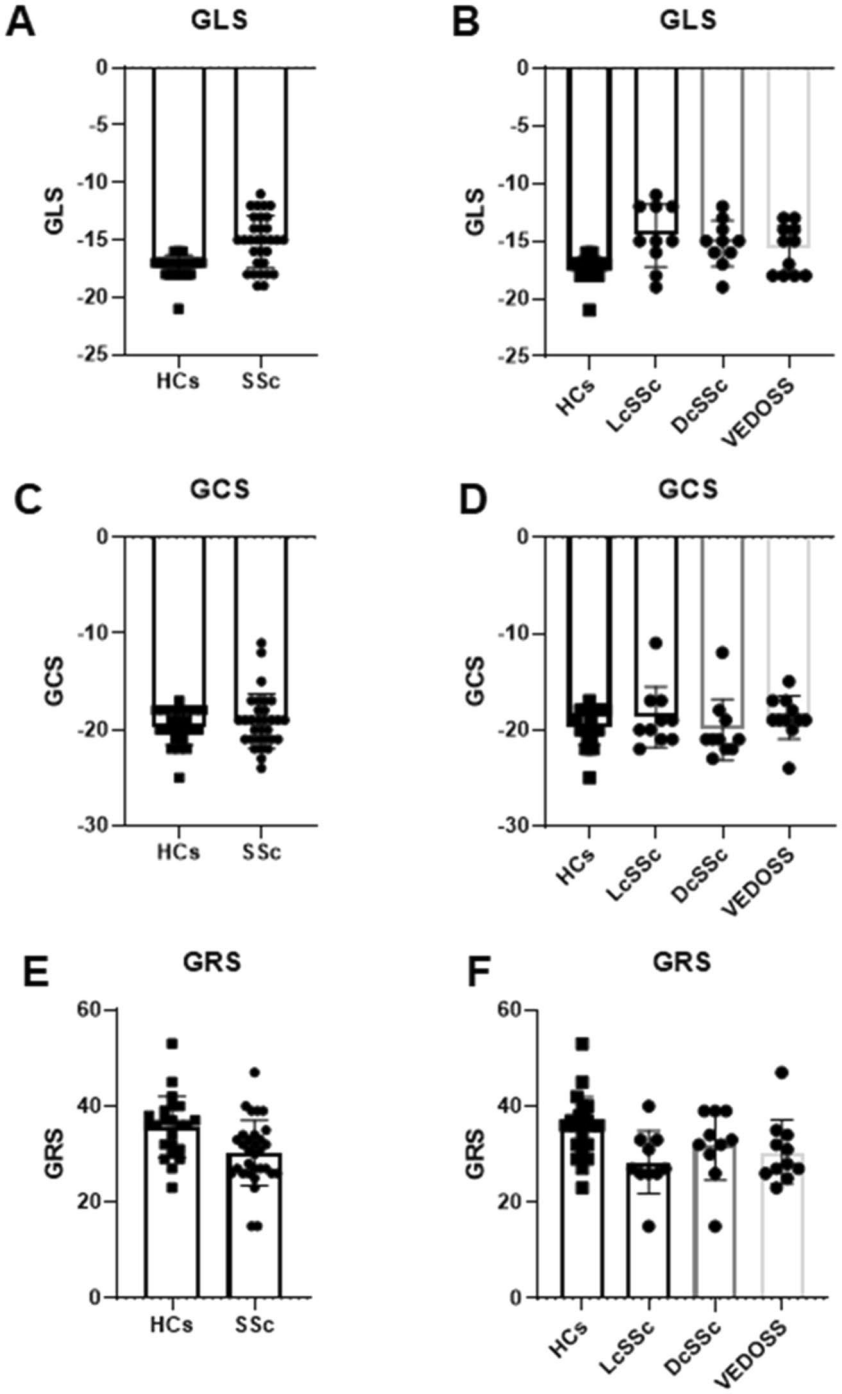
Graphical representation of strain values distribution. (**A**), (**C**) and (**E**) show the comparison between healthy and overall SSc values. In (**B**), (**D**) and (**F**), strain distribution for subset categorization.

Table 2 showed strain values comparison. Healthy values were significantly different with SSc values in radial and longitudinal analysis (*p*-value 0.004 and 0.0001, respectively). Moreover, per-subset analysis evidenced significant difference between: (i.) all subset values with healthy control group for GLS; (ii.) VEDOSS and lcSSc subsets with healthy control group for GRS (Fig. 1). Conversely, GCS resulted similar to healthy values (*p*-value 0.804) (Table 2).

**Table 2:** Strain analysis.

	Healthy	SSc	*p*-value	VEDOSS	*p*-value	LcSSc	*p*-value	DcSSc	*p*-value
Peak global radial strain (%)	36 ± 6	30 ± 7	0.004**	30 ± 7	0.03*	28 ± 7	0.006**	32 ± 7	0.162
Peak global circumferential strain (%)	− 20 ± 2	− 19 ± 3	0.804	− 19 ± 2	–	− 19 ± 3	–	− 20 ± 3	–
Peak global longitudinal strain (%)	− 17 ± 1	− 15 ± 2	0.0001**	− 16 ± 2	0.005**	− 15 ± 3	0.0001**	− 15 ± 2	0.0001**

SSc: Systemic sclerosis; VEDOSS: very early diagnosis of systemic sclerosis; LcSSc: Limited cutaneous Systemic Sclerosis; DcSSc: Diffuse cutaneous Systemic Sclerosis; p: *p*-value.

*Significant test; *p*-value less than .05.

**Significant test; *p*-value less than 0.01.

Lastly, strain values distribution resulted similar among different subset (GLS: *p*-value 0.557; GCS: *p*-value 0.138; GRS: *p*-value 0.335).

### Correlation of strain values with clinical data and interpretative/predictive evaluation

Tables 3 showed Pearson test for parametric correlation. Notably, GLS showed a significant correlation with PASP (r: 0.410; *p*-value: 0.027), with a significant association in a linear regression analysis (b: 1.81, 95% CI 0.222–3.407, *p*-value 0.027).

**Table 3:** Pearson’s correlation.

	GLS (%)		GCS (%)		GRS (%)	
	r	*p*-value	r	*p*-value	r	*p*-value
Age	− 0.04	0.831	0.056	0.764	0.008	0.965
BMI (kg/m^2^)	0.136	0.466	0.061	0.745	− 0.148	0.427
Years of diseas	− 0.009	0.962	0.127	0.497	− 0.024	0.898
PASP (mmHg)	0.410*	0.027*	0.231	0.228	− 0.207	0.282
SCTC-DI baseline	0.206	0.267	0.098	0.6	− 0.154	0.409
mRSS baseline	− 0.129	0.504	− 0.026	0.894	0.159	0.41
LVEDV/BSA (ml/m^2^)	0.181	0.329	0.438*	0.014*	− 0.28	0.127
LVSV/BSA (ml/m^2^)	0.029	0.878	− 0.015	0.938	− 0.012	0.948
LVEF (%)	− 0.232	0.208	− 0.652**	0.0001**	0.362*	0.046*
MyoMass/BSA (g/m^2^)	0.544**	0.002*	0.419*	0.019*	− 0.317	0.083
RVEDV/BSA (ml/m^2^)	0.075	0.688	0.287	0.118	− 0.194	0.296
RVEF (%)	− 0.08	0.67	− 0.47**	0.008**	0.232	0.21

r: coefficient of Pearson; BMI: body mass index; PAP: pulmonary artery pressure; SCTC-DI: Scleroderma Clinical Trial Consortium Damage Index; mRSS: modified Rodnan Skin Score; LVEDV: left ventricular end-diastolic volume; BSA: body surface area; LVSV: left ventricular systolic volume; LVEF: left ventricular ejection fraction; MyoMass: myocardial mass; RVEDV: right ventricular end-diastolic volume; RVEF: right ventricular ejection fraction; GLS: global longitudinal strain; GCS: global circumferential strain; GRS: global radial strain.

*Significant test; *p*-value less than 0.05.

**Significant test; *p*-value less than 0.01.

GLS correlated also with indexed myocardial mass (r: 0.544; *p*-value 0.002), with a good positive b coefficient in a linear regression analysis (b: 3.168, 95% CI 1.313–5.024; *p*-value 0.002).

GCS positively correlated with indexed LVEDV (r: 0.438; *p*-value: 0.014), confirming a significant relation also in a linear regression analysis (b: 1.99, 95% CI 0.439–3.541; *p*-value 0.014).

Both GCS and GRS correlated with LVEF (r: − 0.658; *p*-value: 0.0001) (r: 0.362; *p*-value: 0.046). Both variables showed a predictive association with LVEF when categorized in preserved and reduced EF (GCS: OR: 1.973, 95% CI 1.088–3.58, *p*-value 0.025; GRS: OR: 0.809, 95% CI 0.655–1, *p*-value 0.05).

Finally, only GCS showed a negative correlation with RVEF (r: − 0.47; *p*-value: 0.0001), with a predictive association when categorized in preserved and reduced RVEF (OR: 1.8, 95% CI 1.08–3.11, *p*-value 0.024).

Interestingly, no correlations were founded with continuous STCT-DI or mRSS (r: − 0.036; *p*-value 0.852).

### Pericardial involvement

Pericardial effusion and/or thickness occurred in 9 patients (29%). Significant association was found between pericardial involvement and disease accrual damage (OR: 9.6, *p*-value 0.01).

GLS showed lower values in patients with pericardial involvement (− 16 ± 2% vs − 14 ± 2%, *p*-value 0.016). Conversely, no differences were highlighted in GCS and GRS (− 19 ± 3% vs − 19 ± 2%, *p*-value 0.51; and 29 ± 7% vs 32 ± 6%, *p*-value 0.269; respectively).

### Association between CMR findings and disease accrual damage.

Disease accrual damage occurred predominantly in dcSSc subset, even without significant difference between different subsets, in our cohort.

Among different variables, GRS and GLS showed a significant association with accrual of damage both in a univariate (GLS: HR: 1.36, 95% CI: 1.01–1.82, *p*-value: 0.045; GRS: 1.47, 95% CI 1.09–1.98, *p*-value: 0.029) and multivariate association including all univariate significant predictors (GLS: HR: 1.47, 95% CI 1.09–1.98, *p*-value: 0.011; GRS: 1.22, 95% CI 1.04–1.43, *p*-value: 0.013) (Table 4).

**Table 4:** Regression analysis for association with damage accrual.

	All (n. 31)	
	HR (95% CI)	*p*-value
**Univariate association**		
*Baseline characteristics*		
Age (years)	1.04 (0.97–1.1)	0.27
BMI (kg/m^2^)	1.07 (0.91–1.26)	0.42
PASP (mmHg)	1.05 (0.997–1.11)	0.066
SCTC-DI (baseline)	0.97 (0.74–1.27)	0.82
mRSS (baseline)	1.04 (0.96–1.13)	0.36
*CMR*		
LVEDV/BSA (ml/m^2^)	1.03 (0.98–1.08)	0.325
LVSV/BSA (ml/m^2^)	1.01 (0.93–1.1)	0.755
LVEF (%)	0.95 (0.86–1.05)	0.322
MyoMass/BSA (g/m^2^)	1.03 (0.99–1.07)	0.168
RVEDV/BSA (ml/m^2^)	1.02 (0.98–1.07)	0.355
RVEF (%)	0.94 (0.91–1.1)	0.967
Pericardial Involvement	2.1 (0.62–7.03)	0.231
*CMR-feature tracking SSc subsets*		
Peak Global Radial Strain (%)	1.18 (1.02–1.36)	0.029*
Peak Global Circumferential Strain (%)	0.82 (0.58–1.15)	0.254
Peak Global Longitudinal Strain (%)	1.36 (1.01–1.82)	0.045*
**Multivariate association**		
Peak Global Radial Strain (%)	1.22 (1.04–1.43)	0.013*
Peak Global Longitudinal Strain (%)	1.47 (1.09–1.98)	0.011*

BMI: body mass index; PAP: pulmonary artery pressure; SCTC-DI: Scleroderma Clinical Trial Consortium Damage Index; mRSS: modified Rodnan Skin Score; CMR: cardiac magnetic resonance; LVEDV: left ventricular end-diastolic volume; BSA: body surface area; LVSV: left ventricular systolic volume; LVEF: left ventricular ejection fraction; MyoMass: myocardial mass; RVEDV: right ventricular end-diastolic volume; RVEF: right ventricular ejection fraction; SSc: systemic sclerosis.

*significant test; *p*-value less than 0.05.

**Figure 2:**
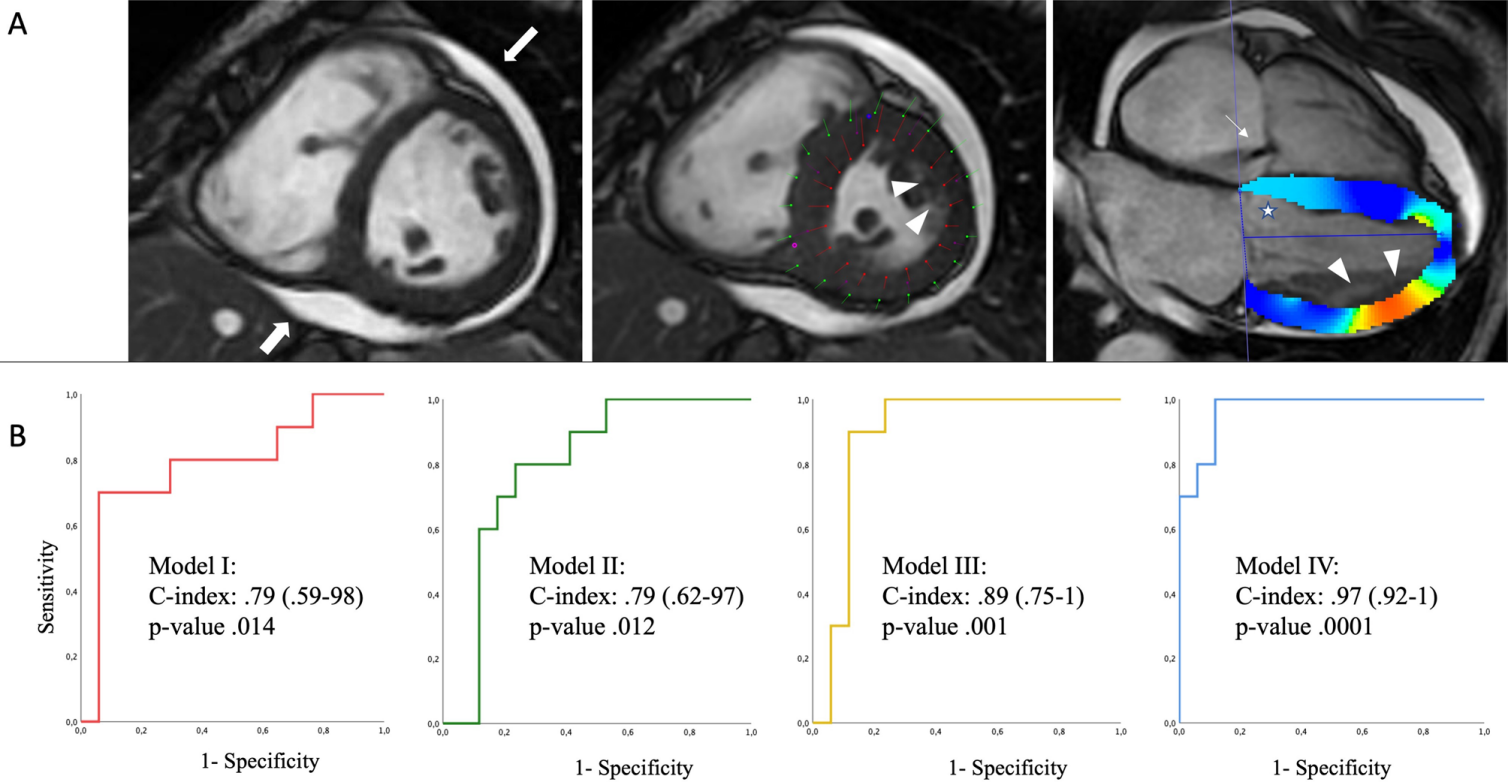
In panel (**A**), a SSc-HD. On the right, color map derived from CMR-TT analysis: darker blue color shows higher deformability, while lighter blue color (white star) (slight) or yellow-to-red color (moderate-to-severe) show areas of low deformability. Sequentially, SSFP cine images acquired in diastolic and systolic phases show moderate pericardial effusion (thick white arrows), with lower longitudinal deformability mainly evident in antero-lateral segment (with arrowheads). In central image, boundary points representation of CMR-TT analysis: epicardial and subendocardial deformability respectively represented in green and red lines-and-dots. Thin white arrow highlight slight tricuspid regurgitation. In (**B**) panel, ROC analysis for Model I, II, III and IV. Model I (conventional clinical score): red line. Model II (Model I + cardiac volumes): green line. Model III (Model II + pericardial involvement): yellow line. Model IV (Model III + strain values): blu line. The highest C-index is reached by the Model IV including both pericardial and strain analysis. SSc: systemic sclerosis; HD: heart disease; CMR: cardiac magnetic resonance; TT: tissue tracking; SSFP: steady-state free precession.

Four model were evaluated, resulted all statistically significant. Model I (conventional clinical score) showed a C-index of 0.79 (95% CI 0.59–0.98, standard error (SE) 0.1, *p*-value 0.014). Model II (Model I + cardiac volumes) did not significantly increase the performance, with a C-index of 0.79 (95% CI 0.62–0.97, SE 0.09, *p*-value 0.012). Once added pericardial involvement, Model III (Model II + pericardial involvement) reached a C-index of 0.89 (95% CI 0.75–1, SE 0.07, *p*-value 0.001). Finally, best C-index was reached by Model IV (Model III + strain values) (0.97, 95% CI 0.92–1, SE 0.03, *p*-value 0.0001) (Fig. 2).

## Discussion

This historically prospective study included naïve patients with a recent diagnosis of SSc (i.e., patients fulfilling the ACR/EULAR 2013 classification criteria in less than 1 in less than 1 year). Our analysis revealed some relevant findings:

(i) Strain analysis by CMR-TT allows to identify an early cardiac involvement in SSc patients when compared with healthy control group;
(ii) GLS only shows a prevalent involvement in all SSc subset;
(iii) Strain correlates with the indices of global cardiac function (i.e. volumes and EF), but not with indices of disease as SCTC-DI and mRSS;
(iv) Early myocardial involvement in SSc patients identified by strain analysis, may be associated with the progression of the systemic damage, recognizing the strain as a possible predictor of damage accrual, independently from standard damage indices.

### SSc-HD, early myocardial damage, and systemic progression

SSc is an autoimmune disorder with a high impact on daily activity and a poor prognosis when associated with a multivisceral involvement. Its heterogeneous clinical presentation leads to uncertainty about disease outcomes and the development of clinical conditions with a poor prognosis.

Beyond an early diagnosis, clinical challenging remains, considering the objective difficulties in determining the extent and activity of the disease, and stratifying potential future complications and identifying individualized treatment.

These objectives remain scarcely applicable to the routine clinical approach, although the identification of early systemic damage remains mandatory for an adequate stratification of the disease.

Among the typical systemic manifestations of SSc, a multi-level involvement of the cardiovascular system is characteristic. This aspect is of considerable interest. Cardiac involvement, indeed, is indicative of a more aggressive pathology, although asymptomatic in 70% of cases^21^. Furthermore, the heart is the key organ of the cardiovascular system that shows a complex fractal organization; this aspect offers the possibility of identifying the heart as a potential target for an early systemic involvement identification and as predictor of clinical aggression and evolution.

CMR imaging is today an uncontested diagnostic tool in the identification of heart diseases, due to the high capability in defining cardiac morphology and its functional characteristics^22–25^. Moreover, scientific attention has recently shifted from the global systolic function to the myocardial deformability components by the strain analysis, which offers the concrete opportunity to identify a precocious impairment of heart function in several diseases and to define specific pattern of myocardial damage^26,27^.

Our results mirrored previous experiences highlighting the ability of strain to discriminate between healthy volunteers and patients with SSc complicated by myocardial involvement^28,29^.

Strain patterns may vary in HD, underscoring the continuum of the disease process.

In particular, GLS is the most sensitive to the presence of myocardial disease^30^. Also in our case series, GLS is reduced in all subsets when compared with the healthy group, despite the preserved LVEF. These findings may confirm the ability of strain in timely unmasking an occult myocardial involvement in SSc since the early stage of disease as in our cohort, thus suggesting its use in clinical practice.

Radial strain also resulted impaired, even most likely related to a more pronounced tethering with longitudinal fibers. No radially oriented fibers are disposed indeed within the myocardium.

Similar mechanism of damage can resulted in localized ischemia which can induce impairment of radial and longitudinal patterns although the preservation of circumferential strain^31,32^.

Conversely, progression of disease results in other-layers dysfunction, leading to a reduction in circumferential strain^33^.

In this regard, GCS only showed a predictive validity on both LVEF and RVEF, reflecting a higher capability in identifying a transmurality of myocardial damage^34^.

Taking together these observations, a better comprehension of all strain values' mechanical aspects therefore results necessary to reach a more profound knowledge of HD^35^.

### SSc-HD and damage accrual

Ability of strain to stage the myocardial damage also translates into the ability to predict systemic disease progression independently from standard damage indices (i.e., SCTC-DI and mRSS), as cardiac involvement is related to a more aggressive systemic disease regardless of heart-related mortality, as shown by Hung et al.^21^.

This consideration finds confirmation in the association analysis between cardiac strain indices and systemic damage accrual, identifying both GLS and tethered GRS as predictors of systemic disease progression.

Finally, some critical considerations should be added in our results.

Combination of pericardial involvement and strain analysis showed the best predictive model performance, since pericardial disease in SSc is known to be predictor of poor prognosis^36,37^.

Caution should be exercised in non-contrast evaluation of pericardium. Pericarditis sicca indeed does not show significant pericardial effusion; therefore, lack of contrast media may not allow an adequate visualization of active forms of pericarditis^38^. However, different case series have already shown a high prevalence of pericardial effusion and chronic forms of pericarditis in SSc, which are typically associated with sclerotic phenomena and pericardial thickening^17,39^.

Moreover, these results derived from the analysis of unenhanced CMR, becoming highly-advantageous considering the rate of kidney involvement in SSc patients^40,41^.

### Limits

This study showed several limits which would reduce the generalisation of the results: (i.) this is a retrospective analysis of CMR examination; (ii.) the analysis was conducted on a relatively small sample size, which implies potential selection bias even if SSc is a rare disease; (iii.) this is a single-centre study, even though the advantages in terms of specimen homogeneity and offered more opportunities to optimize the follow-up studies; (iv.) other CMR indices (i.e. precocious ischemia due to microvascular involvement or myocardial fibrosis by LGE) were not evaluable, however it should be specified that current protocol approved by the Ethics Committee did not foresee any contrast media administration.

## Conclusion

SSc is an aggressive autoimmune disorder with a high clinical impact.

Strain analysis by CMR-TT may show a high capability both in identifying early cardiac involvement and stratifying its clinical aggressiveness, regardless of the standard damage indices and CMR contrast-dependent biomarker.

Strain analysis and unenhanced evaluation of pericardial involvement therefore could help in better identifying a disease subset associated with a poor prognosis, with a potential capacity to guide a timely treatment, even if more studies are needed to fully clarify this issue.

### Institutional statement

All experimental protocols were approved by our Local Ethics Committee (Abruzzo Health Unit 1).

### Informed consent

Informed consent was obtained from all subjects.

## Data Availability

Data are available on request.

## Abbreviations

SSc: Systemic sclerosis
dcSSc: Diffuse cutaneous sclerosis
lcSSc: Limited cutaneous sclerosis
VEDOSS: Very early diagnosis of systemic sclerosis
HD: Heart disease
CMR: Cardiac magnetic resonance
TT: Tissue tracking
ACR: American college of rheumatology
EULAR: European league against rheumatism
LGE: Late gadolinium enhancement
GLS: Global longitudinal strain
GCS: Global circumferential strain
GRS: Global radial strain
SCTC-DI: Scleroderma Clinical Trial Consortium Damage Index
mRSS: Modified Rodnan Skin Score
PASP: Pulmonary Artery Systolic Pressure
LV: Left ventricular
RV: Right ventricular
EDV: End-diastolic volume
BSA: Body surface area
SV: Stroke volume
LVEF: Left ventricular ejection fraction
PAH: Pulmonary artery hypertension
SE: Standard error
